# Characterising and Predicting Benthic Biodiversity for Conservation Planning in Deepwater Environments

**DOI:** 10.1371/journal.pone.0036558

**Published:** 2012-05-11

**Authors:** Piers K. Dunstan, Franziska Althaus, Alan Williams, Nicholas J. Bax

**Affiliations:** The Commonwealth Scientific and Industrial Research Organisation Wealth from Oceans Flagship, Marine Laboratories, Hobart, Tasmania, Australia; National Oceanic and Atmospheric Administration/National Marine Fisheries Service/Southwest Fisheries Science Center, United States of America

## Abstract

Understanding patterns of biodiversity in deep sea systems is increasingly important because human activities are extending further into these areas. However, obtaining data is difficult, limiting the ability of science to inform management decisions. We have used three different methods of quantifying biodiversity to describe patterns of biodiversity in an area that includes two marine reserves in deep water off southern Australia. We used biological data collected during a recent survey, combined with extensive physical data to model, predict and map three different attributes of biodiversity: distributions of common species, beta diversity and rank abundance distributions (RAD). The distribution of each of eight common species was unique, although all the species respond to a depth-correlated physical gradient. Changes in composition (beta diversity) were large, even between sites with very similar environmental conditions. Composition at any one site was highly uncertain, and the suite of species changed dramatically both across and down slope. In contrast, the distributions of the RAD components of biodiversity (community abundance, richness, and evenness) were relatively smooth across the study area, suggesting that assemblage structure (i.e. the distribution of abundances of species) is limited, irrespective of species composition. Seamounts had similar biodiversity based on metrics of species presence, beta diversity, total abundance, richness and evenness to the adjacent continental slope in the same depth ranges. These analyses suggest that conservation objectives need to clearly identify which aspects of biodiversity are valued, and employ an appropriate suite of methods to address these aspects, to ensure that conservation goals are met.

## Introduction

Continental margins – the continental slope and adjacent geomorphic features such as seamounts in depths between approximately 200 and 2,000 m – are the focus of increasing human activity and interest. These areas have a rich and varied biota that is largely undescribed [1] and theories to explain high biodiversity on particular high profile features such as seamounts are evolving rapidly [2], [3], [4], [5], [6], [7]. Deep margins and seamounts feature importantly in biodiversity conservation initiatives, including the commitments made by many nations to establish national reserve networks by 2012 (e.g. in Australia [8]). These initiatives would be furthered by a robust description (or prediction) of biodiversity over broad scales commensurate with that of spatial planning. Reliable descriptions and predictions of biodiversity are a key step in developing and testing hypothesis on the distribution of biodiversity in the deep sea.

Continental margins support industrial-scale demersal fisheries [9], [10], and are a ‘resource frontier’ for oil and gas extraction [11] and mining of high value and ‘high-tech’ metals [12]. Human activities are predicted to expand in the deep sea in the near term [11] in response to declines in shallow water natural resources and, increased pressure on terrestrial resources and rapidly developing technology sectors. It can be expected that much of this activity will be on continental margins and seamounts in depths <2000 m because this is the deep limit of known fishery resources, and is where the extraction of deep-sea hydrocarbon and mineral resources will immediately be most cost-effective. While the impacts of anthropogenic activities on deep benthic ecosystems are thought to be variable in extent and persistence [11], there is a particular need to understand the possible consequences for deep benthic fauna of margins and seamounts. Human impacts (e.g. from bottom fishing) on areas supporting large, slow-growing benthic fauna may be dramatic [13], [14], [15], [16], [17] and long lasting [18], highlighting the imperative to include un-impacted ecosystems of deep margins and seamounts in the considerations of conservation and fisheries management.

Recent research on the continental margins has examined the role of habitat heterogeneity in shaping benthic communities [19], and documenting differences in megafaunal richness, composition and biomass between seamounts and adjacent continental margin [2], [3], [4], [5]. However, understanding the distribution of biodiversity over broad areas in the deep sea has remained an elusive goal because of the difficulty and high cost of biological sampling. Surveys are few, and they typically yield low sample numbers with low sampling density. Surrogate-based approaches that predict the distributions of particular taxa or groups of taxa using more available environmental parameters provide an attractive means of leveraging off the available biological data. Several predictive approaches have been developed [20], [21], [22], [23], [24]; several have been applied to map the distribution of stony corals in the deep sea [25], [26]. Surrogate-based predictive approaches are increasingly powerful as environmental data sets for large areas become more finely resolved and freely available, e.g. the World Ocean Atlas [27] and the CARS climatology for the southern hemisphere [28], [29].

Predictive mapping is based on developing an understanding of the relationships between species distributions and their environment. Prediction uses environmental parameters to constrain the predicted attributes of biodiversity over broad areas, using the observed relationship between environmental parameters and species presence and/or abundance data from local surveys. These relationships are correlative, they do not imply causation and identifying “drivers” for biodiversity requires the careful interpretation of the patterns found to generate hypotheses to describe the distribution of biodiversity. There is no single “right” way to describe or model biodiversity distributions – different hypotheses or management questions require different approaches, and each method of analysis describes different attributes of biodiversity. It is incumbent on scientists to describe the richness and complexity of patterns in biodiversity and not imply a false precision by using only one attribute (eg. species richness). We suggest that a synergistic description of biodiversity using multiple complementary approaches is preferable to using a single metric that may miss useful information and lead to misleading confidence in the maps produced.

In this paper we apply three predictive methods, all developed within the last decade, to map different attributes of biodiversity (the distributions of single species, species richness, and beta diversity) on a deep (∼200–1500 m) and complex continental margin south of Tasmania, Australia, using an extensive environmental data set. This is the first analysis to explore the similarities and differences between patterns of different attributes of biodiversity in a deep water setting. We evaluate the environmental drivers correlated with invertebrate megabenthos by progressively examining ecological patterns in single species distributions, community structure, and community composition over a relatively large area of margin (∼5,500 km^2^). We discuss how the predicted patterns can be used to assist in managing marine biodiversity.

## Methods

### 3.1 Field Survey

**Figure 1:**
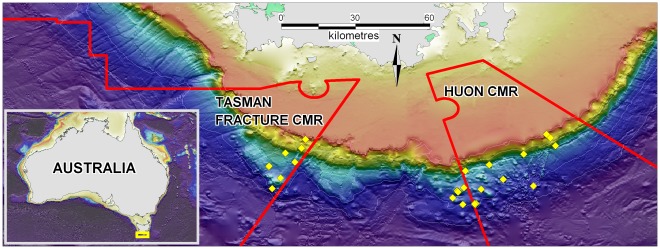
Overview of 39 sample locations from the continental margin south of Tasmania (yellow diamonds). Also shown are the boundaries of the Tasman Fracture and Huon Commonwealth Marine Reserves (CMR) (red lines) and depth contours (white lines): 200 m, 500 m, 1000 m, 1500 m and 2000 m (GA 2009). Inset shows regional location of study area.

Samples of megabenthos (fauna > = 5 mm in size retained by 20 mm stretched cod end meshes; [30] ) were collected with an robust epibenthic sled [31] from the continental margin south of Tasmania during a survey aboard Australia’s Marine National Facility vessel *Southern Surveyor* in 2007 (SS200702) (Figure 1). All biological sampling was conducted with appropriate permits from Australia’s Department of Environment and Heritage, the Australian Fisheries Management Authority and the Australian Animal Ethics Committee. The area studied is a topographically complex continental margin, approximately 150 km in length and characterised by a narrow, steep escarpment-like continental slope with two fields of adjacent seamounts. The slope consists of sediment terraces interspersed with bedding planes of tertiary sedimentary rocks that are exposed in canyon walls and on steep slopes; the seamounts are cone-shaped remnants of extinct volcanoes with peaks in ∼700–1400 m depths, typically with ∼200–300 m elevation and base areas of 1 to ∼20 sq. km. Most sampling sites are within the Tasman Fracture and Huon Commonwealth Marine Reserves (CMRs) declared in 2007 (Figure 1). Of the 39 samples used in analyses, 17 were taken from the continental slope 4 from the upper slope (200–500 m depth) and 13 from the mid-slope (700 m and 1200 m depth) – and 22 were taken from the upper flanks of adjacent seamounts (>700 m depth; Figure 1; Table 1). Replicate samples were taken on four of the seamounts, and on the mid-slope (see Table 1). The sled sample distances varied between 134 m and 2086 m with an average distance of 709 m.

**Table 1:** Details of megabenthos samples from the continental margin south of Tasmania, Australia.

Area	Site	# samples	Target/peak depth (m)	Sampling depth range (m)	Fishing history	# spp identified
Upper slope	Huon	1	200	180–237	Unfished	78
	Huon	1	400	370–410	Unfished	64
	Tasman Fracture	1	200	200–220	Unfished	75
	Tasman Fracture	1	400	410–450	Unfished	59
Mid-slope	Huon	7	1000	800–1030	Unfished	166
	Tasman Fracture	1	1000	800–880	Unfished	50
	Tasman Fracture	4	1200	1100–1200	Unfished	58
	Tasman Fracture	1	1200	1050–1230	Fished	52
Seamount	Dory Hill	1	1054	1100–1200	Unfished	44
	Hill U	5	1083	1100–1160	Unfished	91
	Hill Z15	1	1052	1100–1350	Unfished	41
	Hill Z16	1	1004	1100–1300	Unfished	58
	Little Mongrel	2	1057	1200–1200	Unfished	37
	Little Sister	1	1073	1240–1270	Unfished	58
	Mini Matt	5	1120	1120–1380	Unfished	63
	Z9	1	949	1020–1100	Unfished	41
	Pedra	5	714	730–1000	Fished	56

Continental slope samples were represented the 200, 400, 1000 and 1200 m depth horizons, while the seamount samples were taken from the upper flanks. Fishing history based on logbook records of demersal trawling.

Megabenthos collections were identified to species level by museum experts, principally concentrating on 6 major taxa – sponges, octocorals, echinoderms, decapods, molluscs and ascidians but also including other taxa if they could be identified to species (e.g. some stony corals, and brachiopods). A total of 493 species was identified from the 39 samples; these taxa are included in further analyses and descriptions. Of these 493 species, 47 were caught on the shelf at depths <200 m (47 species). Full details of the surveys, sampling tools, taxonomic processing and fishing history of the survey area are provided in Althaus [17] and Williams [18].

### 3.2 Environmental Data

Two extensive data sets were used to characterise the physical environment of the seafloor in the study area: the CARS climatology [28], [29] for physical oceanography and the MARS sediment database ([32]; http://www.ga.gov.au/oracle/mars/index.jsp) for seabed substratum composition. The MARS sediment database comprises sediment records from over 40,000 samples from around Australia that are used to generate four interpolated sediment layers (% carbonate, % sand, % gravel and % mud) using the inverse distance weighted method [33], the standard interpolation methods used by Geoscience Australia. The sediment layers (ie. % sand, % gravel and % mud) must to sum to 100. Percent carbonate is the carbonate content of seabed sediments expressed as a weight percentage. CARS data provided 10 oceanographic variables (mean and standard deviation of temperature, salinity, oxygen concentration, and phosphate and nitrate levels). The means are the annual means and the standard deviations are the standard deviation of the data from that mean, giving the annual standard deviation. Both sets of data provide temporally integrated, interpolated data layers at 0.01^o^ (1km) grid square resolution. In some cases the environmental data are linearly correlated, particularly the mean values for depth, temperature, oxygen, nitrate and phosphate (Figure S1). However, the standard deviations of the CARS climatologies are not linearly correlated with depth, but show more complex patterns, with distinctive peaks at intermediate depths, suggesting the presence of intermittent deep water currents. The values for carbonate, mud, sand and gravel are completely uncorrelated with the CARS data.

We also included the sample depth and a variable to indicate if a sample location had been commercially fished using bottom trawls or was unfished based on interpretation of the mapped logbook data recorded by the Australian Fisheries Management Authority at 0.1^o^ resolution (*sensu* 16).

### 3.3 Single Species Distributions

The distributions of 8 of the most frequently occurring species (occurring in more than 15 samples) were modelled using aggregated boosted regression trees (ABT; [20]). The 8 selected species represented all 5 phyla with common species: Echinodermata (*Ophiactis abyssicola, Ophiomyxa* MoV5486), Crustacea (*Munida isos, Goreopagurus poorei*), Cnidaria (*Solenosmilia variabilis, Desmophyllum dianthus*), Porifera (*Farrea cf. occa*) and Brachiopoda (*Jaffaia jaffaensis*). ABT’s are a flexible regression tool that can be used to fit environmental gradients to species presence/absence data and then estimate the probability of presence of species across a gridded area characterised by environmental data. ABT’s are derived from boosted regression trees [34] but use multiple tree fits to obtain an average prediction, rather than selecting a single “best” boosted tree. Aggregated boosted trees give consistently more accurate predictions than single boosted regression trees [20]. To prevent over-fitting, the ABT’s were pruned using 10-fold cross-validation to estimate the optimal number of trees to describe the data. The best prediction is then obtained by aggregating the predictions of each of the 10 boosted regression trees.

### 3.4 Beta Diversity

Patterns of beta diversity (compositional turnover) were modelled and predicted using generalised dissimilarity modelling (GDM). GDM is a distance based approach that fits non-linear models based on environmental predictors to ecological distances calculated from a distance measure. The percentage ‘deviance explained’ provides an overall index of how well the spatial distribution of predictor data layers explains variability in the response data [21]. The magnitude of the fitted function for each predictor indicates the total amount of compositional turnover associated with that predictor [35]. By default, GDM calculates dissimilarity between sites as Bray-Curtis distance of presence/absence data [21]. A GDM model based on the same suite of environmental covariates used for the ABTs was fitted to our biological distance matrix to identify the covariates that best described the observed patterns in beta diversity, and to extrapolate the patterns of beta-diversity to each grid square across our study area (*sensu* [21]). Patterns were visualised in a RGB colour map where cells with very different colours are predicted to be highly dissimilar. This visualisation applied multi-dimensional scaling (MDS) to the predicted compositional dissimilarities between grid squares, assigning the first three MDS axes to red, blue and green (RBG) to translate the scores on the first three MDS axes for mapping [21]. We compared the GDM model result with the representation of the differences in species composition between samples in a 2-dimensional non-metric multidimensional scaling (nMDS) ordination of their Bray-Curtis dissimilarities, based on untransformed abundance data [36] that does not account for environmental gradients. We could then compare the predicted dissimilarities in the modelled beta-diversity between the sampling positions with the observed dissimilarities between the species compositions of the samples.

### 3.5 Multispecies Abundances

We used a recently developed method of analysing and predicting multispecies responses to environmental gradients using rank abundance distributions (RAD) [22], [23]. The rank abundance distribution for each sample is broken into three components: total abundance, species richness and relative species abundance. We define the likelihood of the RAD distribution for site *i* as.
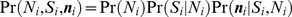
(1)


Total abundance (*N_i_*) is the sum of the abundances of all the species in each sample (*i*) excluding those species that cannot be identified to individuals (eg corals). Species richness (*S_i_*) is the total number of species in a sample. Relative species abundance (***n*** *_i_*) is the proportion of each species in a sample and is a multivariate response represented by a vector of length (*S_i_*). Breaking the RAD into three components allows each component to be modelled separately, significantly simplifying the modelling process. *S_i_* is truncated so that it cannot exceed the total number of individuals; *N_i_* is used as a covariate of *S_i_* (*S_i_* is conditional on *N_i_*). *N_i_* and *S_i_* are also used as covariates for the multivariate response of relative abundance (***n*** *_i_*) so that ***n*** *_i_* is conditional on *N_i_* and *S_i_*. We scale both *N_i_* & *S_i_* by the area sampled by the sled to control for differing effort. To simplify spatial prediction and interpretation of relative abundance, evenness (η_i_) is defined as the derivative of the slope of the RAD curve at the most abundant species [22], [23]. Models are fitted to each component using the environmental gradients as covariates and the fitted models are predicted spatially using the gridded environmental data sets. The models were selected using a combination of forward selection and the Akaike Information Criterion (AIC) as described in [22]. Polynomial terms and interactions were tested.

## Results

### 4.1 Overview of Megabenthos Collected

Echinoderms were the most speciose group with 28% of all identified species, closely followed by sponges (27%) and then cnidarians (17%). In terms of biomass (total catch weights of the 39 samples) this order was reversed Cnidarians (41%, mainly octocorals and scleractinians) dominated, followed by sponges (25%) and echinoderms (20%). Species distribution over the samples was typical for deep-sea samples with a high number of singletons; 207 species (43%) were caught only once. An additional 25 species where caught in only one of the 39 samples analysed here but they were also collected on the shallower shelf. The percentage of singletons was slightly lower on seamounts (24%) compared to the slope (29% each, upper and mid-slope). The upper slope was the richest area with 295 species; it was dominated by sponges (Porifera), both by number of species and by total biomass. This phylum was rarer in the deeper depths of the mid-slope and on seamounts, where corals (Cnidaria) and echinoderms dominated the number of species and total biomass. Species of all other phyla were relatively evenly distributed over the three areas.

### 4.2 Single Species Distributions

**Figure 2:**
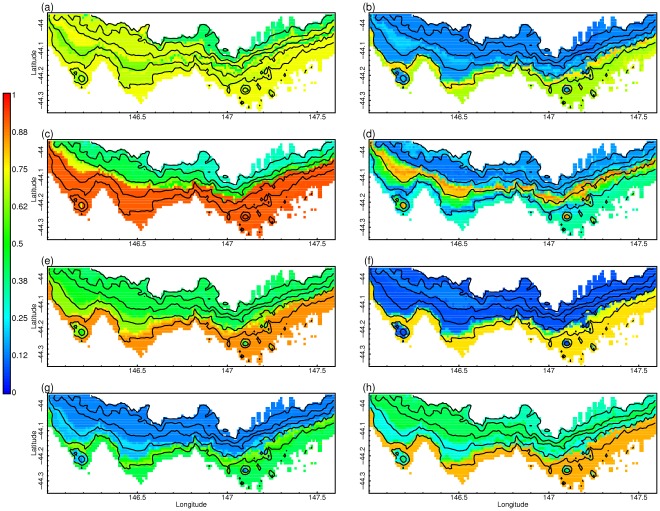
Distributions of eight of the most frequently occurring species of megabenthos from the deep continental margin south of Tasmania, Australia. The distributions are shown for: (a) *Ophiactis abyssicola*; (b) *Ophiomyxa* MoV5486; (c ) *Munida isos*; (d) *Goreopagurus poorei* (e) *Solenosmilia variabilis*; (f) *Desmophyllum dianthus*; (g ) *Farrea cf. occa*; (h) *Jaffaia jaffaensis.* Bathymetry contours are 200 m, 400 m, 600 m, 1000 m, and 1200 m.

The most frequently occurring species were found at mid-slope and seamount depths where the majority of samples were taken. Despite all being relatively deep dwelling species, each had distinctly different predicted patterns of distribution and occurrence (Figure 2). All the species have relatively low probabilities of occurrence, indicating that sampling repeatedly in the same location will not always detect the same species. Several of the species show changes in likelihood of occurrence between 600 m and 1000 m. All of the common species are correlated with oceanographic covariates (e.g. temperature, phosphate and nitrate) but some species show only weak relationships with physical habitat covariates (i.e. mud, sand, gravel; Table 2). Most conspicuously, 6 species (*M. isos, J. jaffaensis, S. variabilis, F. occa, Ophiomyxa* and *D. dianthus*) are strongly (positively) associated with nitrate concentration (Table 2). The standard deviation of phosphorus concentration was negatively associated to 5 species (*J. jaffaensis, S. variabilis, F. occa, Ophiomyxa* and *D. dianthus*). Variation in temperature (i.e. standard deviation) was important for 3 species, the relationship is strongly positive for the 2 crustaceans (*M. isos* and *G. poorei*) and strongly negative for the echinoderm *O. abyssicola*. Temperature variation was high in the 500–1000 m depth range that includes the summits of the larger seamounts, and lower at shallower and deeper depths. Generally, variation (i.e. the standard deviation) of oceanographic variables is not strongly correlated with depth (Figure S1). Surprisingly, there were no strong associations of common species with oxygen concentration or salinity, with the single exception of *G. poorei*. There were few strong responses of common species to geological covariates, with only *O. abyssicola* showing a strong positive response to gravel, *S. variabilis* a strong negative response to mud, *F. occa* positively associated with mud, and *Ophiomyxa* a positive response to sand. *Jaffa jaffaensis*, appeared to have a preference for intermediate values of mud, but is a brachiopod known to be associated with coral-matrix (K. Gowlett-Holmes (CSIRO) pers. comm.). The presence of only 1 of the common species (*O. abyssicola*) appeared to be strongly negatively affected by fishing. The absolute probabilities of presence are low for even the common species (Figure 2), suggesting that even the “common” species will be relatively infrequently found.

**Table 2:** Boosted Regression tree summaries of 8 frequently occurring species of megabenthos from the deep continental margin south of Tasmania, Australia.

Covariate	*Munida isos*		*Ophiactis abyssicola*		*Jaffaia jaffaensis*		*Goreopagurus poorei*	
depth	**15.50**	(+)	0.65		2.56		1.73	
fishing	0.19		**14.35**	(–)	4.16		0.73	
gravel	6.49		**17.85**	(+ peak)	3.57		3.07	
mud	3.83		1.38		**16.26**	(+ peak)	4.66	
Nitrogen	**15.53**	(+)	0.60		**29.78**	(+)	2.47	
Nitrogen sd	0.30		0.98		1.17		5.84	
Oxygen	8.55		0.70		0.98		0.73	
Oxygen sd	4.95		5.60		0.62		**21.05**	(–)
Phosphate	0.01		0.31		4.88		1.92	
Phosphate sd	0.91		0.31		**19.21**	(–)	0.61	
Salinity	3.82		**11.30**	(+)	3.47		**24.37**	(–)
Salinity sd	8.04		0.65		0.34		6.53	
sand	0.40		3.90		2.09		3.82	
Temperature	**19.81**	(–)	0.76		3.28		5.98	
Temperature sd	**11.66**		**40.67**	(–)	7.62		**16.50**	(+ peak)
AUC	0.805		0.603		0.722		0.772	

Covariate	*Solenosmilia variabilis*		*Farrea* cf. *occa*		*Ophiomyxa* MoV5486		*Desmophyllum dianthus*	
depth	1.62		4.35		2.11		4.19	
fishing	0.48		0		0		0	
gravel	4.26		0.87		1.86		4.60	
mud	**25.66**	(–)	**10.01**	(+)	0.24		1.11	
Nitrogen	**33.54**	(+)	**46.06**	(+)	**30.10**	(+)	**44.75**	(+)
Nitrogen sd	2.60		1.21		0.73		0.24	
Oxygen	0.95		2.29		3.17		2.15	
Oxygen sd	0.52		0.26		0.01		0.03	
Phosphate	6.75		**10.43**	(+)	4.08		6.41	(+)
Phosphate sd	**11.45**	(–)	**11.26**	(-)	**26.08**	(–)	**30.45**	(–)
Salinity	0.43		4.28		0.67		0.26	
Salinity sd	0.14		1.42		2.67		0.30	
sand	0.46		0.89		**25.85**	(+)	2.85	
Temperature	**10.53**	(–)	6.24		1.03		1.00	
Temperature sd	0.62		0.42		1.40		1.68	
AUC	0.879		0.736		0.816		0.894	

The summaries show the percent variation explained by each covariate for each species. Variables with greater than 10% contribution are in bold and the sign of their relationship shown. The AUC summary statistic summarising model prediction accuracy is also given for each species; sd = standard deviation).

### 4.3 Beta-diversity

Modelling the turn-over in community composition (beta diversity) showed the spatial distribution of 12 covariates (out of 14) explained 52.4% of the variability in Bray-Curtis dissimilarity between sites for 493 species. The analysis suggests that there is a high degree of variability in the species composition between two samples because of the low probabilities of occurrence of any single species. When the environmental distance is zero, the predicted dissimilarity at the intercept is 0.59, indicating that only 41% of species will be shared in samples taken in the same environmental space. The total amount of species turn-over associated with the individual environmental predictors, as indicated by the amplitudes of the fitted functions, was relatively low (<1) for all predictors. Salinity and depth contributed most strongly to explain compositional turn-over (magnitude of fitted function 0.96 and 0.95 respectively), followed by mud (0.55), sand (0.48), phosphate (0.43) and standard deviation in salinity concentration (0.40). The contributions of temperature, nitrate (standard deviation and mean) and oxygen concentration were moderate (0.25, 0.17, 0.11 and 0.11, respectively), while the lowest relative contributions were made by the variability in oxygen (0.004) and carbonate concentration (0.06). Temperature and phosphate did not contribute to the final model.

**Figure 3:**
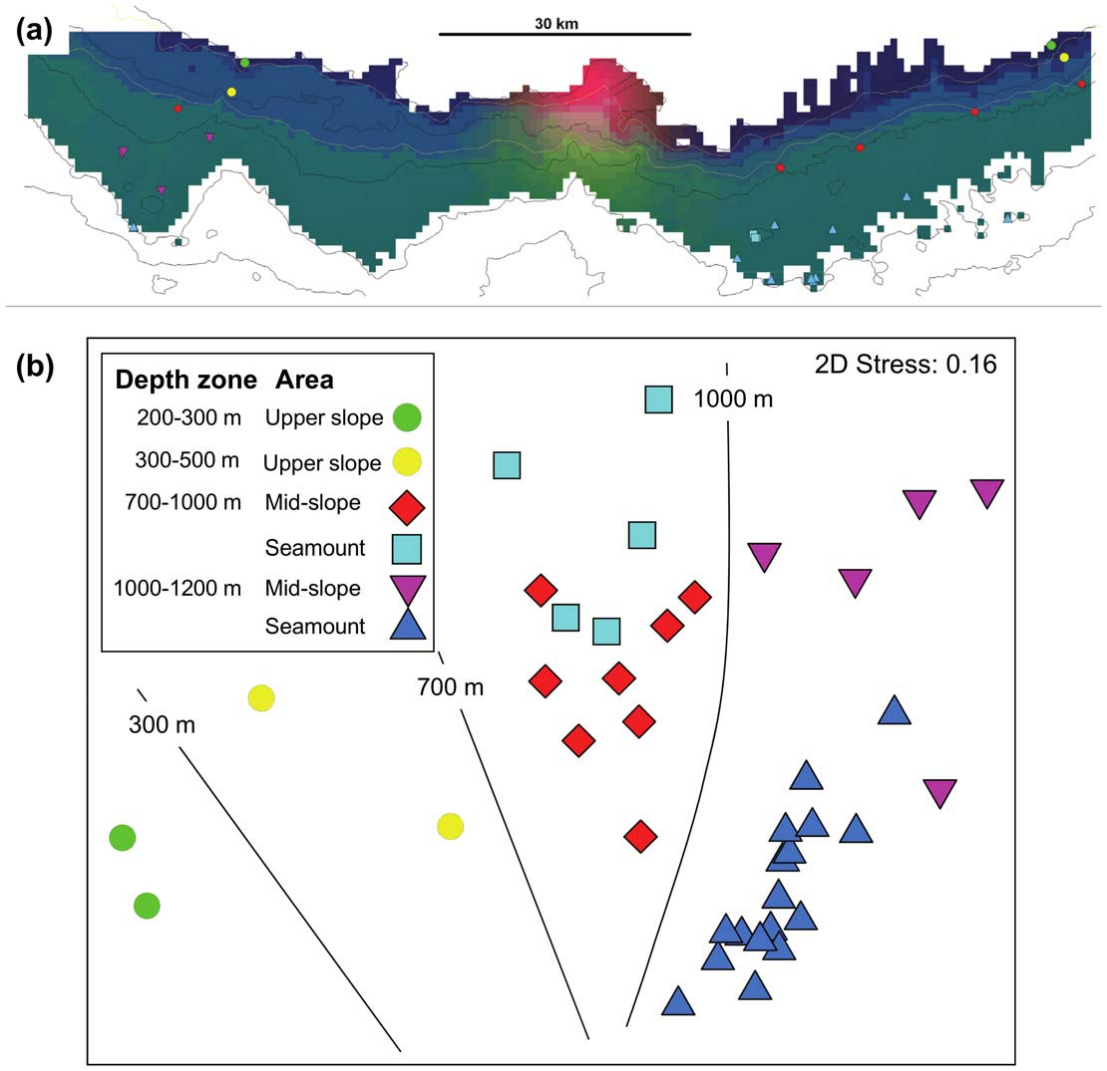
Representations of the spatial pattern in megabenthos composition on the continental margin south of Tasmania, Australia. A visualisation of showing beta-diversity (a), derived by applying multi-dimensional scaling to the compositional dissimilarities predicted by a GDM model fitted to epibenthic sled sample data (symbols) (areas of similar colours are predicted to have similar fauna composition; bathymetry contours: 200 m, 500 m, 1000 m, 1500 m, 2000 m); and (b) an MDS ordination of the Bray-Curtis dissimilarity between samples based on species abundance data, coded by bathome and margin vs seamount.

Using the fitted model we created a prediction grid of the patterns of compositional turn-over across the entire study area (Figure 3a). Spatially, the predicted beta-diversity changes with increasing depth. There are clear changes in beta-diversity (colour) predicted around 300 m and 700 m depth (Figure 3a). Similar colours represent areas that have more similar species composition, noting that the most similar sites only share 40% of the same species. This separation by depth is clearly represented in the nMDS of the samples (Figuere 3b). The second apparent split in the nMDS, separating samples from the 700–1000 m depth zone from deeper samples was only represented by a very subtle change in beta-diversity, barely discernible in the colour scheme, at the 1000 m depth contour, (Figure 3a). In addition to the depth pattern there is a gradual change in beta-diversity from both east and the far west towards the centre of the study area characterised by a higher proportions of mud; no samples were collected in this central region (Figure 3a). The spread in the nMDS of the sample points of the single shallow seamount sampled (Figure 3b) demonstrates the high variability between samples from one location, i.e. where the environmental distance is zero.

### 4.4 Multispecies Abundances

**Figure 4:**
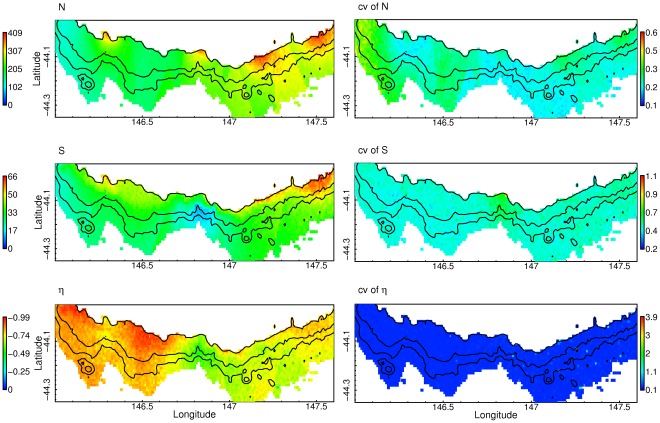
Predictions of megabenthos diversity from the deep continental margin south of Tasmania, Australia. Plots show total abundance (N) and the coefficient of variation of N; species richness (S) and the coefficient of variation of S; species evenness (η) and the coefficient of variation of evenness. A value of zero for the coefficients of variation indicates no variation around the mean prediction and a value of 1 indicates that the standard error is equal to the mean.

Patterns of community structure (ie the distribution of multispecies abundances) show surprisingly little variation over the scales considered, the major variation being along the slope in contrast to down slope for single species (Figure 4). Total abundances in the samples from all depths was only associated with carbonate (Table 3), generating a distinctive response from west to east of increasing total abundance apparent in the predictions of N (Figure 4). The fitted values also indicate that swept area was only weakly positively associated with total abundance. This can be interpreted as a significant underlying variation in the patterns of abundance that cannot be described by the environmental covariates alone. Interactions between previous fishing effort and the sampling method may have contributed to this uncertainty.

**Table 3:** Fitted values and standard errors for the analysis of rank abundance distributions of megabenthos from the deep continental margin south of Tasmania, Australia.

	Coefficient	(SE)	AIC
*Total Abundance (N)*			
intercept	−1.36	(3.19)	526.68
log(area)	0.089	(0.20)	516.25
Percent carbonate	0.073	(0.03)	512.25
Theta	1.73	(0.37)	
*Species Richness (S)*			
Intercept	3.53	(0.03)	321.95
log(area)	0.25	(0.04)	–
log(N scale)	0.31	(0.08)	313.13
N scale	0.13	(0.08)	285.33
Nitrogen	−2.84	(0.95)	274.44
Sand	−0.10	(0.06)	273.55
Sand:Nitrogen	2.68	(0.93)	268
Theta	148.86	(160.54)	
*Species Evenness (η)*			
Intercept	1.25	(0.02)	4373.82
N scale	1.17	(0.21)	4369.01
N scale^2^	−1.48	(0.48)	4322.46
S scale	−0.63	(0.1)	4281.64
N scale^3^cube	0.619	(0.31)	4279.91
S scale^2^	0.37	(0.09)	4263.82

A negative coefficient indicates that the term reduces the response (ie N, S, evenness) and a positive term indicates an increase in the response. (N scaled = scaled for area sampled).

Species richness showed strong responses to log N (scaled for area sampled by the sled) and raw N scaled for area sampled by the sled, giving a power relationship between S and N. Species richness increases with N, but eventually plateaus irrespective of increases in N as the total species pool or large aggregations are sampled (Table 3). There may be several abundant species that can achieve high densities and dominate a particular area. Richness also decreased with increasing nitrate concentration. Depth and nitrate are strongly correlated, indicating a similar pattern of decreasing richness with depth observed in the raw data.

There was no direct response of species evenness to any of the environmental covariates analysed, and patterns are a function of richness and abundance (i.e. only richness and abundance are covariates). Species evenness was more evenly distributed in the deeper part of the study area (Figure 4), although evenness was generally high overall. Evenness indirectly responds to the environment through the responses of richness and abundance. This results in a consistent pattern of evenness throughout the study area.

## Discussion

### 5.1 Ecological Patterns in Continental Margin Megabenthos

A striking feature of the predicted distributions of megabenthos living on the deep continental margin off southern Tasmania is the variability of individual species. All eight common species we examined are inhabitants of mid-continental slope depths, but each had a different distribution as defined by its probability of presence in 1km grid squares in the 5,500 km^2^ prediction area. Thus, apart from an overall association between species distribution and mid-slope depths, there appear to be no common patterns, with each species responding to the environment in a different way. The overall species-level pattern is one of differences rather than similarities. There are several strong associations of species to covariates that are related to depth, especially nitrogen concentration, but it is clear that depth is not the only driving environmental gradient. All 8 common species responded to at least one aspect of environmental variation which, assuming that substratum properties have remained stable, implies that they have responded to variation in oceanography. This is consistent with the idea that these species have responded over long time scales to variability in physical properties, including nutrient availability, conferred by the interactions of currents overlaying the study region. At this location, megabenthos is within the Sub-Tropical Convergence (STC) zone and influenced by multiple currents. Inter-annual variation in seasonally-changing near-surface water alters water column productivity as flows of the nutrient-poor Leeuwin Current and East Australia Current [37] replace subantarctic surface water. Water masses in greater depths, subantarctic mode water (between ∼250 and 600 m) [38] and antarctic intermediate water (AAIW) below this, are seasonally persistent; however, there are episodic intrusions of [colder, nutrient rich] subantarctic water [39], complex flows in AAIW [40], and outflow from the Tasman Sea, that generate a dynamic oceanographic climatology over the continental margin [41].

While nitrate is important for many species, it is strongly correlated with depth, phosphate, and temperature (Figure S1). The relationships for all analyses are complex (e.g. the interaction between sand and nitrogen for species richness; Table 3). Identifying the ecological drivers amongst correlated variables is only possible through inference when using regression type approaches. Intuitively, there are a number of covariates that would have direct causative influences on biota (e.g. currents, particulate organic carbon, calcite, distribution of rocky or biogenic substrata), but these are difficult or expensive to measure and validate. We have limited our analysis to covariates that can be directly measured across the region of interest (e.g. temperature and sediment properties) to reduce the possible bias that may occur when using covariates that come from other complex models (e.g. currents). For sessile species such as the habitat forming stony coral *S. variabilis*, the presence of rock for stable attachment is critical to its distribution. Unfortunately, there is currently no fine scale (<1 km) information on seabed hardness to allow predictions at the large scales relevant to individual marine reserves or our study area, i.e. typically 100's to 1000s of square kilometres. Hence, probabilities of presence for all species need to be interpreted acknowledging that there are some missing covariates. Sampling methods will also influence the observations - there is no guarantee that sampling has not added variation to the patterns observed here. For example, sled samples at such depths will not sample all species equally, and will under-represent fauna associated with the cracks, crevices and vertical walls found in rocky habitats. Furthermore, sled samples integrate across habitat types along transects, and the sampled biomass may exceed the sampling capacity of the sled when benthic biomass is high, e.g. on biogenic coral substratum. Care needs to be taken to minimise these sources of variation during sled-based surveys. Visual survey offers an alternative to physical samples from sleds [17], [42], but still have biases in observed species abundances and distributions.

Patterns in beta diversity, based on all 493 species found in the survey, were consistent with the expectation of a highly variable change in species composition with space indicated by the single species analysis. Beta-diversity within the same site (i.e. locations within the study area defined by identical covariates) can be estimated from the intercept term of the GDM model. Its high value of 0.59 shows that 59% of species are predicted to vary from sample to sample within environmental space characterised by the same covariates, i.e. that dissimilarly may be very high between adjacent sites. This is consistent with patterns seen in the single species and RAD analyses, indicating that composition changes rapidly, even within a local area, but that community structure, defined by rank abundance and richness, does not. High species turnover (>70%) was observed on the flanks of a single seamount [43], reflecting both fine scale variability in environment (e.g. substrate) and change across a bathymetric gradient. Also consistent with the patterns seen for single species, beta-diversity changed broadly with depth. These compositional changes with depth were characterised by the rapid loss of shallow species (particularly Demosponges) below the shelf break in about 300 m depth, and their replacement with fauna including corals characteristically found in deeper water. Some shallow slope and deep slope sites share no common species despite being separated by horizontal distances of only a few kilometres; the upper slope (200–500 m) and deep seamounts (>1000 m) have no common species, representing a shift from sponge dominated assemblages to deep water stony coral dominated assemblages. There is a large area in the middle of the region that is quite distinct from the rest of the seamounts where patterns appear to be driven by high concentrations of mud. This region is also apparent in some of the single species models, the RAD analysis, as well as in the beta-diversity distribution.

In contrast to single species patterns, analyses of multispecies rank abundance distributions (RAD) are remarkably invariant. They show a gradual decrease in abundances and species richness with depth, but the trends do not result in large differences over the depth range sampled. There is also an east-west gradient apparent where both abundance and species richness trends from low to high moving from west to east. Species evenness does not show any discernible pattern with depth, but also shows a decreasing trend from west to east. Given the high variation shown by individual species, it is surprising that the distributions of total abundance, richness and evenness show so little variation in contract to patterns seen in similar depth ranges in Western Australia [23]. This leads to an expectation that species composition is highly variable, but that the species richness of continental margin megafauna varies little between locations. Further, these patterns in aggregate imply that there is a limit to the total abundance, species richness and evenness that remain unchanged as community composition changes. The long tail in species abundances is common across all sites and depths. Deep continental margin species may be limited by the supply of particulate organic carbon (POC; [44], [45]) below the mixing layer and the patterns seen here may be a reflection of that process.

### 5.2 Conservation Planning in Deepwater Environments

Our results show the potential strengths, and limitations, of this suite of methods for marine biodiversity conservation planning in areas such as deep continental margins where biodiversity distributions are typically poorly defined. In many locations where marine reserves are planned, design decisions have to be made in the absence of complete information on biodiversity composition and distribution, and predictive tools can help fill that gap. Thus, where management objectives are defined by conservation outcomes that protect the maximum numbers and diversity of species in reserves it becomes advantageous to incorporate a suite of tools to assist in reserve design, including beta-diversity and the RAD distributions. These methods collectively suggest that on the Tasmanian margin there is no single location that typifies the patterns of biodiversity of this region, rather that large areas across a range of depths are needed to maximise the representation of species within reserves.

These underlying patterns in biodiversity distributions would not be apparent if a single method was used – patterns are best analysed from multiple viewpoints. The tools we have used here are appropriate to understanding large scale patterns of biodiversity and biogeography. Considered at these scales, there is little to distinguish slope from seamount habitat, and it is apparent that composition is extremely variable. However, these methods are not appropriate for fine scale analysis and do not capture the high abundances of particular species such as the important habitat-forming coral *S. variabilis* which occurs on seamounts but not on the slope [17], [18]. To quantify patterns in abundances, particularly in colonial animals, other tools and statistical methods are needed (e.g. [9]). Management planning for biodiversity conservation typically focuses both on individual ‘iconic’ species, and biodiversity as a ‘whole’, indicating the need for a suite of analytical methods that describe biodiversity distributions from several viewpoints. Our multiple-methods approach may not maximise the protection of a single priority species, but it has considerable potential to complement single species approaches for biodiversity conservation in areas such as continental margins where biological data are sparse.

### 5.3 Conclusion

Using a suite of analyses to examine the distribution of benthic megafauna provides new insights into the ways the environment may influence their structure on a deep and complex continental margin. Species presence may be highly variable and assemblages in the same environmental space may be quite dissimilar. Despite high variability in species composition, RAD measures of community structure appear to vary slowly over the spatial domain sampled. Thus, the total species richness appears to be limited and largely constant for any depth band despite the low probability of collecting many of the same individual species in replicate samples. The high underlying variability in the distributions of species in the study area suggests that management objectives for reserves need to clearly consider which components of biodiversity they aim to protect.

## Supporting Information

Figure S1 **Biplots of all 15 covariates considered for inclusion in predictive models.** Each cell shows the relationship between two covariates, listed on the diagonal.
(PNG)
