# Supplementary figures and images for: Characterising and Predicting Benthic Biodiversity for Conservation Planning in Deepwater Environments
Source: PLoS One. 2012 May 11;7(5):e36558. doi: 10.1371/journal.pone.0036558 (PMC3350518; doi:10.1371/journal.pone.0036558)

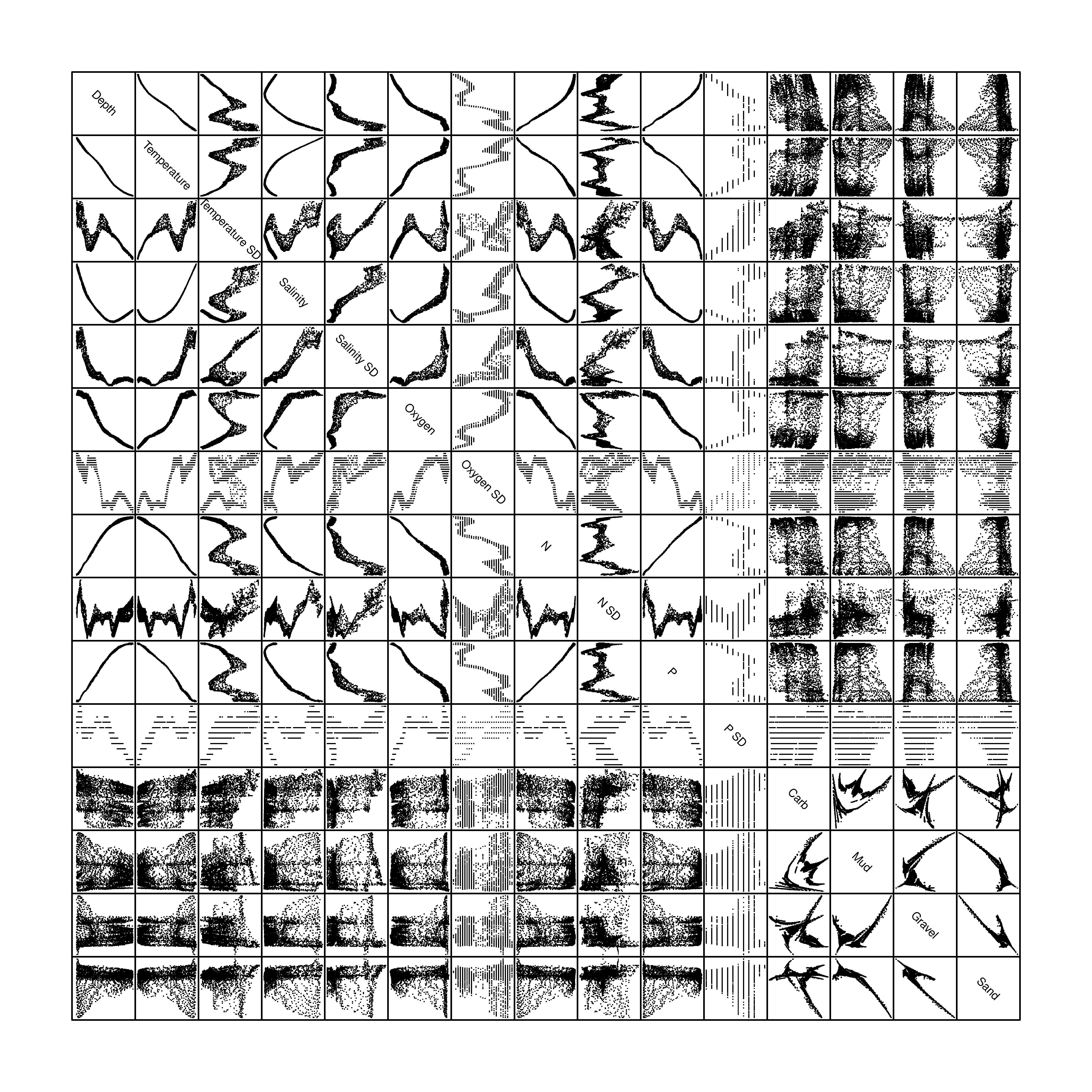

Supplement: Figure S1 — Biplots of all 15 covariates considered for inclusion in predictive models. Each cell shows the relationship between two covariates, listed on the diagonal. (PNG) [file pone.0036558.s001.png]
